# Isolated Abducens Nerve Palsy in the Setting of Isolated Sphenoid Sinusitis: A Case Report

**DOI:** 10.7759/cureus.46993

**Published:** 2023-10-13

**Authors:** Richard I Suarez, Michaela B Polmann, William M Portnoy, Emily Quintero, Kebir Bedran

**Affiliations:** 1 Herbert Wertheim College of Medicine, Florida International University, Miami, USA; 2 Otolaryngology, Baptist Health South Florida, Miami, USA; 3 Family Medicine, Baptist Health South Florida, Miami, USA; 4 Hospital Medicine, Baptist Health South Florida, Miami, USA

**Keywords:** sixth cranial nerve palsy, isolated sixth nerve palsy, isolated sphenoid sinusitis, isolated abducens nerve palsy, sphenoid sinusitis

## Abstract

The cranial nerves (CNs) are responsible for multiple functions, including extraocular mobility, facial sensation and movement, hearing, mastication, tongue movement and sensation, and swallowing. Beyond these vital roles, they can also demonstrate importance in their diagnostic value. Isolated or combined palsies provide insights into potential localizations and various underlying etiologies, including stroke, tumor, and infections that may guide further neurological evaluation. CN VI, the abducens nerve, singularly innervates the lateral rectus muscle, which is responsible for the abduction of the eyes. Despite its long anatomic trajectory, making it susceptible to intracranial injury, an isolated abducens nerve palsy is extremely rare. The most common clinical presentation includes headache, diplopia, and the inability to abduct the afflicted eye. This case report introduces a 71-year-old female with a medical history of malignancy and pancytopenia who presented to the emergency room with complaints of ear pain and swelling and subsequently developed diplopia secondary to unilateral CN VI palsy. Magnetic resonance imaging (MRI) revealed isolated sphenoid sinusitis for which she was clinically asymptomatic. She was treated with a regimen of ampicillin-sulbactam, an oral anti-inflammatory agent, and a tapered course of methylprednisolone with a rapid and complete resolution of the abducens nerve palsy and sinusitis. Acute isolated diplopia is an unusual neurologic condition prompting the need for rapid and thorough investigation. Although exceedingly rare and infrequently cited in the literature, isolated abducens nerve palsies secondary to sphenoid sinusitis should be entertained in the differential diagnosis of this presentation.

## Introduction

The cranial nerves (CNs) play vital roles in day-to-day human activities; familiarity with their anatomic courses and function can also be instrumental in their diagnostic value. Isolated or combined palsies can provide clues to various underlying etiologies, such as stroke, tumors, infections, and other medical conditions, prompting further neurological evaluation. CN VI, the abducens nerve, solely innervates the lateral rectus muscle, which is responsible for the abduction of the eyes. It originates within the brainstem near the fourth ventricle exiting via the pontomedullary junction, which is both caudal and medial to the facial nerve (CN VII) and vestibulocochlear nerve (CN VIII) [1]. It then traverses through the subarachnoid space crossing the petrous part of the temporal bone inside Dorello’s canal, which is a fibrous sheath that serves as an anchor for the nerve, before entering the dura and subsequently the cavernous sinus [1]. Within the sinus, it travels with multiple other CNs (III, IV, V1, and V2) and the internal carotid artery. Before exiting the superior orbital fissure to innervate the lateral rectus, the nerve courses along the sphenoidal fissure. Along this path, the nerve is particularly vulnerable to injury because it has the second longest intracranial course of all the CNs [1]. When this nerve is injured, patients tend to present with unilateral abduction dysfunction with associated diplopia-binocular and horizontal [2-3]. In addition to diplopia, patients may report pain with eye movements, pain around the eyes, and/or headache [4-6]. Neuropathy involving different CNs and other neurological findings have also been reported with CN VI palsies [4-6]. Acute diplopia is an unusual and potentially concerning symptom for stroke, tumor, or other intracranial processes, emphasizing the need for its elucidation [7]. Although abducens nerve palsy is exceedingly rare (incidence rate of 11.3/100,000 cases), it is the most common cranial neuropathy reported in adults presenting with diplopia, largely among the elderly population and can be recurrent [2,6-7].

When evaluating suspected CN injuries, the differential diagnoses can be vast. The most common cause of cranial neuropathy, including isolated CN VI palsy, is due to a microvascular insult [6-7]. Any intracranial process that applies direct pressure on the nerve or stretches the nerve, such as a tumor, vascular lesion, or trauma, can result in nerve injuries. Indirect pressure, such as that from edema, hydrocephalus, or pseudotumor cerebri, results in downward pressure on the brainstem and consequent nerve injury as it stretches along the clivus. Other identified causes are decompensation; ophthalmic migraine; immunologic conditions, including multiple sclerosis, myasthenia gravis, and Creutzfeld-Jacob disease; thyroid-related diseases; tumors, such as craniopharyngiomas; infections, such as neurosyphilis; and macrovascular injuries [7-10]. Relatedly, patients with diabetes mellitus and hypertension are also at risk considering their vascular complications. 

Acute or chronic sphenoid sinusitis is an exceptionally rare cause of isolated abducens nerve palsy with few cases reported within the literature. Prior cases of associated unilateral, contralateral, or bilateral palsy uniformly present with at least one symptom commonly attributed to sinusitis, namely, non-specific headaches or notable facial and/or orbital pain [11-15]. Here, we report the case of a 71-year-old female who developed an isolated CN VI palsy as a result of asymptomatic sphenoid sinusitis. With this in mind, the reporting of isolated abducens nerve palsy highlights the need for practitioners from various medical disciplines to be familiar with its clinical presentation, significance of its anatomical pathway, various etiologies, and need for prompt interventions.

## Case presentation

This patient is a 71-year-old female with a significant past medical history of progressive metastatic breast cancer (status post right mastectomy and multiple rounds of chemotherapy), pulmonary embolism (not on anticoagulation), and pancytopenia who presented to the emergency department as suggested by her primary care provider due to significantly low hemoglobin. She also complained of left ear pain. Additional past medical history included chronic obstructive pulmonary disease (COPD) with emphysema predominance, anxiety, liver cirrhosis, osteoporosis, lumbago, osteoarthritis, and a moderate hiatal hernia with gastroesophageal reflux disease (GERD). The patient was evaluated in the outpatient setting prior to arrival and her hemoglobin (Hgb) was 5.6 g/dL, prompting her doctor to refer her to the hospital. Upon arrival, she was found to be afebrile and tachycardic (132 bpm) with a respiratory rate of 18, blood pressure of 124/68 mmHg, and oxygen saturation of 100% on room air. On review of systems, the patient reported left ear pain and palpitations but denied fever, chills, nasal congestion, trauma to the ear, hearing loss, recent swimming, or other exposures to bodies of water. On physical examination, there was swelling and erythema of the left external auditory canal and pinna with no active discharge. Her laboratory studies demonstrated a white blood cell (WBC) count of 4.7 x 10^9^ /L (differential showing 44% neutrophils and 4% bands), Hgb of 6.8 g/dL (reference range: 12.1 to 15.1 g/dL), hematocrit (Hct) of 23.7%, platelets of 46,000/microliter (reference range: 157,000 to 371,000/microliter), and D-dimer of 4.04 mg/L (reference range: 0 to 0.50 mg/L). The remainder of her values were unremarkable, including her creatinine, blood urea nitrogen (BUN), glucose, and liver function tests. Chest radiography and chest computed tomography (CT) scan with pulmonary embolism (PE) protocol showed chronic interstitial changes with a superimposed right middle lung infiltrate and trace bilateral effusion with no evidence of acute PE. Before inpatient admission, the patient received two units of packed red blood cells with improvement of Hgb to 10.5 g/dL and one dose of vancomycin. 

The patient was admitted due to symptomatic anemia and further investigation of left ear pain considering significant past medical history. An infectious disease consultation was placed considering symptomatology pointing to otitis externa in an immunosuppressed patient. The patient reported that she had taken azithromycin for about five days outpatient without symptom relief. Inpatient, she was started on daptomycin (6 mg/kg IV every 24 hours) but had no change in clinical presentation and continued to endorse pain and mild left ear hearing loss. Her antibiotic regimen was changed to ampicillin-sulbactam (3 g IV every six hours). Topical steroid drops and an oral anti-inflammatory agent were also added. Over the next two days, the patient began to have a symptom resolution with decreased pain and reduced swelling and erythema of the left external auditory canal and pinna. Blood cultures and methicillin-resistant *Staphylococcus aureus* (MRSA) nasal screening were negative. 

**Figure 1:**
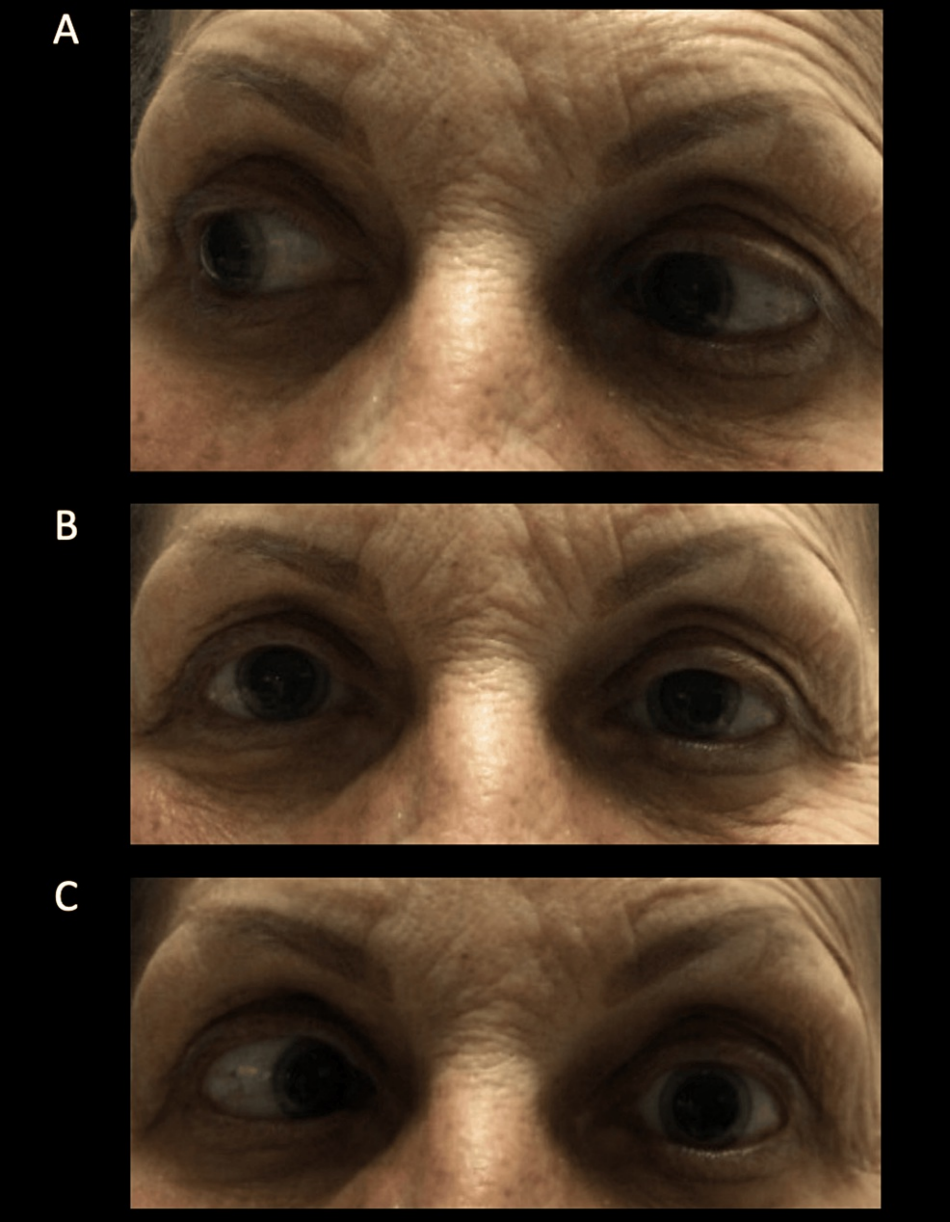
Cranial nerve VI palsy visualization A: Patient is moving her eyes to the right (abduction of the right eye, adduction of the left eye) with no extraocular difficulties noted. B: Patient has her eyes midline. C: Patient is moving her eyes to the left (abduction of the left eye, adduction of the right eye) with noted difficulty with the abduction of the left eye as it remains at midline.

**Figure 2:**
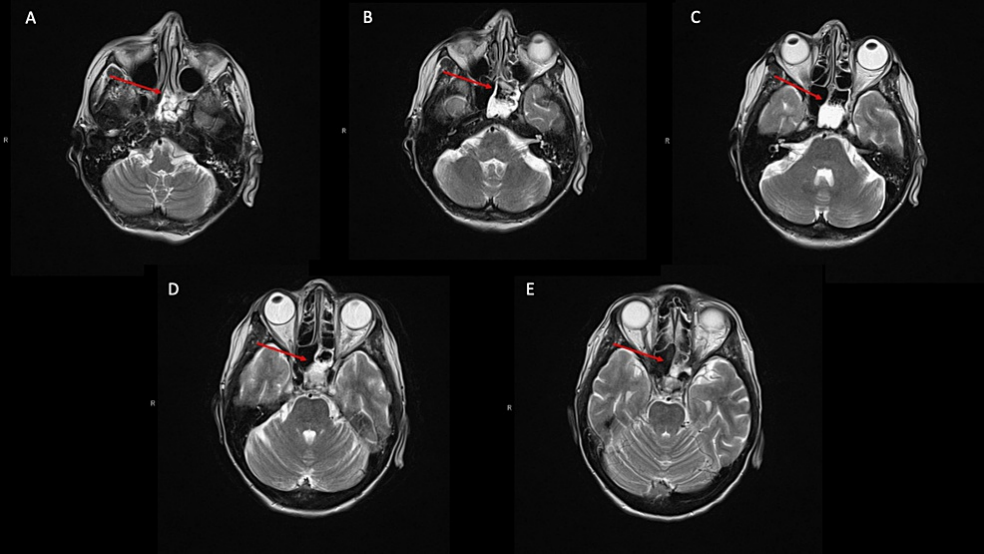
Brain MRI Red arrows denote the abnormal findings. A-E represent the image sequence flow, moving from inferior to superior. Radiology report described no acute intracranial or intraorbital abnormalities seen. Opacification of the left sphenoid sinus consistent with acute sphenoid sinusitis. Minimal small vessel ischemic change and tiny old left cerebellar lacunar infarct. Partial opacification of the left mastoid and minimal opacification of the right mastoid. No thrombosis of a cavernous sinus. Orbits are normal. No compression of the optic chiasm. Thin sections through the orbits demonstrate optic nerves, extraocular muscles, and intraorbital fat to be within normal limits and not compressed.

On day three of the new regimen, despite clinical improvement, the patient began to complain of new-onset left eye discomfort, which she originally attributed to potential trauma via scratching while she was sleeping. Upon neurological examination, the patient was found to have mild left eye diplopia, which resolved with the closing of her eyes, and the inability to abduct her left eye with all other extraocular movements intact (Figure 1). The patient denied headache, nasal congestion, lacrimation, pain, blurry vision, or rhinorrhea but did note a mild pressure sensation around her eyes. Neurology and otolaryngology were consulted to examine for infectious versus vascular (cavernous sinus syndrome) versus malignant process resulting in the isolated CN VI palsy. Brain magnetic resonance imaging (MRI) demonstrated isolated opacification of the left sphenoid sinus without findings of an acute vascular event or signs of metastatic process (Figure 2). 

Considering these findings and the patient’s clinical presentation, she was diagnosed with an isolated, ipsilateral CN VI palsy secondary to acute sphenoid sinusitis. She continued her treatment regimen of ampicillin-sulbactam (3 g IV every six hours) along with a tapered course of methylprednisolone 4 mg for a total of six days. The patient completed her treatment with subsequent resolution of the abducens nerve palsy, sinusitis, and otitis externa/cellulitis. She was eventually transferred to another facility for further evaluation of her pancytopenia.

## Discussion

Although diplopia is a common clinical presentation, particularly in the elderly, abducens nerve palsy is seldom seen, especially in the setting of sphenoid sinusitis. Our patient presented with lateral rectus palsy with painless diplopia and associated mild orbital pressure sensation. Painful diplopia, by contrast, is more concerning and suggests an intraocular or intracranial process, likely traumatic, vascular, neoplastic, or inflammatory in nature [16]. Therefore, it is important to distinguish which type of diplopia is present to guide the diagnostic evaluation. There is no difference in the incidence of isolated CN VI palsy in women compared to men [17]. The mean age of patients who acquire CN VI palsy is 60 years of age [18], so our 71-year-old patient is slightly older than this demographic. On average, patients with CN VI palsy take two and half months to fully recover [18]; our patient recovered at an incredibly fast rate with her extraocular movements restored to baseline within a week of initiation of her treatment. It is important to note that the recovery rate is heavily dependent on the etiology of the palsy. In a study assessing the clinical outcomes of CN VI palsy, those with microvascular etiology typically had a recovery rate of 100%, while those with brain vascular lesions recovered at a rate of only 64%. Those whose etiologies fell into the category of “others,” as in our patient, recovered at a rate of 86.67%. Unfortunately, 17.95% of participants in this study failed to recover [18].

Common causes of CN VI palsy include microvascular disease, brain vascular lesions, neoplasm, and trauma [7-8,18]. In this patient, neoplasm and cavernous sinus thrombosis were certainly among our top differential diagnoses due to her extensive cancer history. Isolated sphenoid sinusitis is quite rare, accounting for less than 3% of all paranasal sinus pathologies [12]. Relatedly, sinusitis is a very uncommon cause of CN VI palsy and, thus, was not initially on our differential. A small number of cases of CN VI palsy with isolated sphenoid sinusitis have been reported before, each presenting with unilateral, contralateral, or bilateral palsy and diplopia. 

Most cases had some variations of associated symptoms, including progressively worsening headaches, often hemi-cranial-disequilibrium, orbital or ocular pain, blurred vision, fever, and/or nasal congestion [11-15]. In addition, some cases required more extensive treatment beyond conservative and antibiotic measures, such as transnasal sphenoidotomy [13,19] or sphenoid drainage [11]. Although our patient did have unilateral left palsy with significant sphenoid sinusitis, our case was unique in that she did not present with any of the typical associated symptoms one would expect with acute sphenoid sinusitis, namely, headaches radiating to the vertex or occiput of the skull. Furthermore, she had a speedy and complete recovery without the need for more drastic interventions, unlike other reported cases. This speaks to the importance of early identification of the underlying pathology and initiation of appropriate therapy, in this case antibiotics and steroids.

## Conclusions

Isolated CN VI palsy is a rare finding, especially when caused by sinusitis. This case demonstrates the importance of a thorough and expedient search for the underlying cause. In particular, the diagnostic value of the CN assessment and the need to keep one’s differential diagnosis broad when evaluating possible etiologies of isolated CN palsies cannot be overstated. Due to its anatomical proximity to the sphenoid sinus, sphenoid sinus pathology should be entertained when considering the differential diagnoses for this unusual clinical presentation. Despite our patient’s advanced age well beyond the norm for a patient with this cranial neuropathy, our patient made a rapid and full recovery of less than one week compared to the average of two and half months and did not require more extensive treatment beyond antibiotic and conservative measures. As such, one may surmise that the initiation of prompt and appropriate targeted therapy could contribute to the early resolution of this cranial neuropathy.
